# High-flow nasal cannula failure: can clinical outcomes determine early interruption?

**DOI:** 10.31744/einstein_journal/2021AO5846

**Published:** 2021-06-02

**Authors:** Milena Siciliano Nascimento, Danielle Eugênia Ribeiro Quinto, Gisele Cristina Zamberlan, Adriana Zamprônio dos Santos, Celso Moura Rebello, Cristiane do Prado

**Affiliations:** 1 Hospital Israelita Albert Einstein São PauloSP Brazil Hospital Israelita Albert Einstein, São Paulo, SP, Brazil.

**Keywords:** Cannula, Bronchiolitis, Risk factors, Respiration, Child

## Abstract

**Objective:**

To evaluate the evolution of clinical outcomes in children with bronchiolitis who used a high-flow nasal cannula, and to determine after long of non-clinical improvement the therapy should be discontinued, and treatment should be escalated to other forms of ventilatory support.

**Methods:**

An observational retrospective study of infants with bronchiolitis who used a high-flow nasal cannula. Patients were divided into two study groups according to success or failure of high-flow nasal cannula therapy, namely the Success Group and the Failure Group. The main demographics and clinical variables were assessed 30 minutes and 6 hours after initiating therapy until removal of the high-flow nasal cannula.

**Results:**

A total of 83 children were studied and 18 children (21.7%) failed therapy. Among subjects with successful therapy, a significant decrease in respiratory rate (p<0.001), and a significant increase in peripheral oxygen saturation (p<0.001) were observed within 30 minutes. The Success Group was significantly different from the Failure Group after 6 hours, for both respiratory rate (p<0.01) and peripheral oxygen saturation (p<0.01).

**Conclusion:**

The absence of clinical sign improvement within 30 minutes and for up to a maximum of 6 hours can be considered as failure of the high-flow nasal cannula therapy. If this time elapses with no improvements, escalating to another type of ventilatory support should be considered.

## INTRODUCTION

The high-flow nasal cannula (HFNC) is widely used for respiratory support in patients with bronchiolitis. The device relies on the non-invasive delivery of high flows of heated, humidified air, with titratable fractions of oxygen, which can generate positive driving pressure, increasing functional residual capacity, and reducing work of breathing.^(^^1^^,^^2^^)^

The use of this therapy has been associated with improved washout of the nasopharyngeal dead space and better mucocilliary clearance, in addition to more accurate oxygen delivery compared with other systems.^(^^3^^)^ The high-flow nasal cannula has been able to improve oxygen saturation (SpO_2_) and is associated with decreased end-tidal carbon dioxide (EtCO_2_) and lower respiratory rates (RR) in children with bronchiolitis admitted to the intensive care unit (ICU).^(^^4^^,^^5^^)^

Initially, the studies aimed to generate protocols to guide titration of the flow. Protocols were created establishing predetermined flows by age,^(^^6^^)^ and others used weight-based flow titration; currently the most often flow varies from 1.0 to 2.0L.kg-^1^.min-^1^.^(^^7^^−^^10^^)^

Although some authors have studied clinical variables, such as heart rate (HR), RR, fraction of inspired oxygen (FiO_2_) and SpO_2_, to determine HFNC therapy failure, there is still no consensus on the maximum acceptable values.^(^^6^^−^^8^^,^^11^^,^^12^^)^ Moreover, there are no studies on the time required to determine HFNC therapy failure, which is well established for noninvasive mechanical ventilation (NIV), both in adults and pediatric patients.^(^^13^^−^^16^^)^

For decades, NIV was used to manage respiratory failure without any understanding of the limits of this therapy, which are currently known. Studies on NIV failure requiring orotracheal intubation highlighted the fact that patients with no short-term improvement (1 to 2 hours) have a higher risk of NIV failure,^(^^13^^,^^14^^)^ and a delay in discontinuing NIV may be associated with increased mortality.^(^^15^^,^^16^^)^ Defining outcome criteria and time frames to stop HFNC therapy is key to ensure the safety of this therapy.

## OBJECTIVE

To evaluate the evolution of clinical outcomes in children with bronchiolitis who used high-flow nasalcannula, and to determine after long of non-clinical improvement the therapy should be discontinued, and treatment should be escalated to other forms of ventilatory support.

## METHODS

### Type and setti,ng of the study

An observational retrospective study was conducted at the Pediatric Intensive Care Unit of *Hospital Israelita Albert Einstein,* through review of pediatric medical records that met the inclusion criteria for the sample design. Since this was a retrospective study, there was no requirement for signing of an informed consent form (ICF).

### Patients

This study enrolled children aged under 2 years with a diagnosis of bronchiolitis, who used HFNC as the first choice for management of respiratory failure, and were admitted to the pediatric intensive care unit (PICU) of *Hospital Israelita Albert Einstein*, in the city of São Paulo (SP), between January 2016 and June 2017.

Bronchiolitis is defined as inflammation of small airways of viral etiology, which affects children aged 0 to 2 years. It progresses with increased mucus secretion and airway edema, leading to airway obstruction of variable intensity.

The variables assessed included age, sex, weight on admission, Pediatric Index of Mortality (PIM) 2, Modified Wood’s Clinical Asthma Score (M-WCAS), and duration of HFNC therapy. To assess the clinical outcome, patients were evaluated for RR, HR, FiO_2_ and SpO_2_, at pre-setup, 30 minutes, 6 hours and HFNC removal.

For data analysis, patients were divided into two groups: Success Group (patients who responded to HFNC therapy) and the Failure Group (patients that required a different type of ventilatory support).

### High-flow nasal cannula failure criteria

The criteria for HFNC failure have not been established yet at the organization where the study was conducted. Thus, failure of HFNC therapy was based on evaluation by the care team, and defined as the need for NIV or invasive mechanical ventilation (IMV).

### High-flow device

For HFNC therapy, one of the two devices available at the pediatric ICU was used: Optiflow™ (Fisher & Paykel Healthcare, Auckland, New Zealand) or Precision Flow^®^ – Vapotherm (New Hampshire, United States of America). The Optiflow™ system was associated with the Babypap^®^ 1150-S blender (Fanem, Guarulhos, SP, Brazil). Optiflow™ Junior 2 nasal cannulas (Fisher & Paykel Healthcare, Auckland, New Zealand) were used in four sizes, as appropriate for each patient, namely: OPT312 Premature, OPT314 Neonatal, OPT316 Infant and OPT318 Pediatric. The Precision Flow^®^ was used with four different cannula sizes: neonate, infant, pediatric small and pediatric. The usage protocol was based on flow titration at 2.0L.kg^−1^.min^−1^ for patients weighing up to 10kg; for patients over 10kg, a flow of 2.0L.kg^−1^.min^−1^ was used for the first 10kg, and 0.5L.kg^−1^.min^−1^ for every kg over 10kg.

### Statistical analysis

Categorical variables were reported as absolute frequencies and percentages. Numerical variables had their distributions verified on boxplots and reported as medians and quartiles, due to asymmetrical distributions and outlying values. The description is provided for all patients and based on failure or non-use of HFNC.

To compare the profile of patients with success or failure of the HFNC therapy, hypothesis testing was used: χ^2^ test, Fisher’s exact test and Mann-Whitney test, as appropriate.

To investigate variations in the values measured over time, mixed models were adjusted considering the dependence between values obtained at different timepoints, for the same individual. Gamma distributions were considered for being the most suitable to continuous symmetrical data. Results were reported as estimated mean values, 95% confidence intervals (95%CI) and p values.

In case of multiple comparisons, p values were corrected using the sequential Bonferroni method.

Analyses were conducted with the software (SPSS), with a significance level of 5%.

This work was submitted to the Research Ethics Committee of the organization for approval and is registered under number 2.316.087, CAAE: 77279317.4.0000.0071.

## RESULTS

Of the 1,749 children admitted during the study period, 363 (20.8%) had a primary diagnosis of bronchiolitis. High-flow nasal cannula was used by 83 children of them. High-flow nasal cannula therapy failed in 18 children (21.7%), of which 5 (6.0%) required mechanical ventilation (MV) and 17 (20.5%) required NIV; four patients required both MV and NIV.

Table 1 shows the main demographics, clinical interventions and late events of all patients, and patients by success or failure of HFNC therapy. There was no difference in demographics between the groups. In respect to the duration of HFNC therapy, patients in the Failure Group used the therapy for a shorter period than patients in the Success Group: 12.8 hours *versus* 56.8 hours (p<0.001).

**Table 1:** Demographics of all patients, and patients who succeeded or failed therapy with high-flow nasal cannula

Variables		Total (n=83)	Group		p value
			Success (n=65)	Failure (n=18)	
Age, months		2.00 (1.00-6.00)	3.00 (1.00-6.00)	2.00 (1.25-3.00)	0.55*
Sex					
	Male	46 (55.4)	39 (60.0)	7 (38.9)	0.18†
	Female	37 (44.6)	26 (40.0)	11 (61.1)	
Weight upon admission, kg		5.70	5.80	4.95	0.11*
PIM 2, severity (0-100%)		0.16	0.16	0.16	0.73*
M-WCAS		4.00	4.00	4.00	0.50*
Time of HFNC usage, hours		4.00 (25.87-70.50)	56.8 (40.00-74.67)	12.8 (8.87-23.69)	<0.001*

Values reported as median (interquartile range) or n (%).

* Mann-Whitney test;

† p values for χ^2^ test.

PIM: Pediatric Index of Mortality; M-WCAS: Modified Wood’s Clinical Asthma Score; HFNC: high-flow nasal cannula.

Table 2 presents the estimated adjusted-model means for RR, FiO_2_ and SpO_2_, comparing timepoints at pre-setup, 30 minutes after setup, 6 hours after setup and HFNC removal, and also comparing the Success and Failure Groups.

**Table 2:** Adjusted mean values and 95% confidence intervals for measurements taken at pre-setup, 30 minutes, 6 hours and upon removal of the high-flow nasal cannula

	Success Group (n=65)				Failure Group (n=18)			
	Pre-HFNC	30 minutes	6 hours	Removal	Pre-HFNC	30 minutes	6 hours	Removal
RR (bpm)	54.6 (52.2-57.1)	48.4 (46.4-50.4)*	45.6 (43.7-47.5)*	39.7 (37.8-41.6)*	59.5 (54.6-64.5)	52.5 (48.5-56.5)	51.9 (47.9-55.9)	59.7 (54.9-64.4)
FiO_2_ (%)	37.1 (33.6-40.6)	31.0 (28.6-33.5)†	28.8 (27.0-30.7)*	24.2 (22.7-25.6)*	34.9 (28.7-41.0)	29.8 (25.3-34.3)	30.9 (27.1-34.6)	33.8 (30.6-37.0)
SpO_2_ (%)	94.1 (92.8-95.4)	97.0 (96.5-97.5)*	97.2 (96.7-97.8)*	97.2 (96.3-98.1)*	94.9 (92.4-97.4)	97.7 (96.7-98.7)	95.6 (94.4-96.7)	92.8 (91.1-94.5)

* p<0.001 *versus* the pre-setup value of the Success Group;

† p <0.01 *versus* the pre-setup value of the Success Group.

HFNC: high-flow nasal cannula; RR: respiratory rate; bpm: breaths per minute; FiO_2_: fraction of inspired oxygen; SpO_2_: peripheral oxygen saturation.

Overall, for patients with HFNC therapy success, there was a significant drop in RR and FiO_2_ as early as within 30 minutes (p<0.001), as well as a significant rise in SpO_2_ within the first 30 minutes of HFNC therapy (p<0.001). Patients with HFNC therapy success were significantly different from those of the Failure Group after 6 hours, for both RR (p<0.01) and SpO_2_ (p<0,01).

## DISCUSSION

The literature shows great evolution in understanding of HFNC therapy in the pediatric population, particularly in infants with bronchiolitis; however, the failure criteria and the need for therapy escalation have not been fully understood yet.^(^^11^^−^^12^^,^^17^^−^^21^^)^

To date, very few studies have investigated clinical variables to determine HFNC therapy failure.^(^^17^^−^^21^^)^ This was one of the pioneer studies establishing a time frame for therapy discontinuation upon absence of improvement, in addition to defining clinical parameters. Determining these parameters and their behavior between initiating therapy and discontinuation improves the safety of HFNC usage, allowing for earlier verification of absence of treatment response and preventing deterioration of the respiratory system.

In this study, patients who failed HFNC therapy showed no clinical improvement with RR and FiO_2_ reduction, and SpO_2_ increase within 30 minutes after initiating therapy, which was seen in subjects who successfully responded. It also showed that it is possible to find a difference in RR and SpO_2_ between patients with therapy success and failure after 6 hours.

Mayfield et al., supported the findings of this study, since they also showed improved RR among patients who responded to HFNC therapy within 1 hour. However, the number of patients in that analysis was much lower than that of the present study: only 8 patients in the failure group.^(^^9^^)^

Other studies have also shown no improvement in RR and oxygen levels in patients who failed the therapy, however without discussing the time until discontinuation.^(^^17^^−^^19^^)^

Although the behavior of parameters like HR, RR and FiO_2_ after HFNC adaptation is related with success or failure of the therapy, there is no consensus regarding how long one should wait before reevaluating the patient, verifying the absence of improvement and discontinuing HFNC therapy. Some studies have shown variable times until discontinuation and escalation to NIV or IMV, including 12.8 hours in the present study, 5.5. hours,^(^^20^^)^ 7 hours^(^^18^^)^ and 14 hours.^(^^20^^)^ This entails some considerations regarding the monitoring of these patients while using HFNC, and shows that verification of non-improvement and escalation to a different therapy can take place earlier.

The findings in this study show patients with HFNC therapy success responded fast (30 minutes) and, 6 hours after onset, there is a significant difference in RR and SpO_2_ between the groups (p<0.01). The results of this study suggest patients who do not respond to therapy after 6 hours, still presenting with tachypnea and requiring FiO_2_ over 30% to reach SpO_2_ >95%, must be escalated to other forms of therapy. Research has shown^(^^17^^,^^18^^,^^20^^)^ therapy discontinuation in time frames longer than 6 hours, and in clinical practice, for some patients, it can take more than 24 hours before a decision is made to discontinue therapy. The findings of the present study, in this sense, can contribute to earlier escalation of the therapy and improved patient safety.

In this study, HFNC therapy failure was observed in 21.7% of cases, of which 5 required orotracheal intubation. The literature shows great variability in the failure rate of high-flow therapy in patients with bronchiolitis (0% to 50%).^(^^17^^,^^21^^−^^23^^)^ Milési et al., when comparing HFNC with nasal continuous positive airway pressure (CPAP) in patients with bronchiolitis, observed a 50% failure rate with HFNC.^(^^21^^)^ The profile of patients in that study in respect to the M-WCAS was very similar to that of patients enrolled in this study. Nevertheless, in the present study, the failure rate was lower than found by Milési et al., 21.7% *versus* 50%.^(^^21^^)^ This can be explained by the difference in initial flow rates between the Milési et al., and the present study, *i.e.* 1.0L.kg^−1^.min^−1^ *versus* 2.0L.kg^−1^.min^−1^.^(^^21^^)^

Franklin et al., in a study comparing HFNC with low-flow nasal cannula in patients with bronchiolitis, observed a 10% failure rate for HFNC, whereas 61% of patients who failed low-flow therapy were successfully rescued after initiating HFNC therapy.^(^^23^^)^ The high variability in the HFNC failure rate is justified by different factors in the literature.^(^^17^^,^^21^^−^^23^^)^ The profile of subjects is one of these factors, since milder cases with a lower distress score have a higher chance of responding to the therapy, whereas more severe cases with a higher distress score, have a higher chance of failure. The location is another factor, when comparing ICUs and inpatient wards. Also, the lack of standardization of the target flow and the use of subjective evaluation methods to determine therapy failure can hinder the comparison of failure rates between the different studies.

This study has the limitations inherent to a retrospective analysis, despite no bias in the HFNC therapy usage, HFNC usage protocol was very well established at the time cases were surveyed. Another limitation is the small number of patients who failed HFNC during the study period. Although 83 children were enrolled, only 18 children failed HFNC therapy, which limited the power of the study. To date, studies looking into risk factors for HFNC therapy failure have shown a similar profile, with 14 children with therapy failure in Betters et al.,^(^^20^^)^ and 8 in Mayfield et al.^(^^9^^)^

## CONCLUSION

In the absence of improvements in clinical signs, such as lowering of the respiratory rate and fraction of inspired oxygen, and rise of the oxygen saturation, high-flow nasal therapy failure can be established as early as 30 minutes, and up to a maximum of 6 hours, after the onset of therapy. After this time has elapsed with no improvements, escalating to a different type of ventilatory support should be considered.
