# Supplementary material for: High-flow nasal cannula failure: can clinical outcomes determine early interruption?
Source: Einstein (Sao Paulo). 2021 Jun 2;19:eAO5846. doi: 10.31744/einstein_journal/2021AO5846 (PMC8225260; doi:10.31744/einstein_journal/2021AO5846)
Supplement: Translation

# Translation: Falha da cânula nasal de alto fluxo: os desfechos clínicos podem determinar a interrupção precoce?

**Authors:** Milena Siciliano Nascimento, Danielle Eugênia Ribeiro Quinto, Gisele Cristina Zamberlan, Adriana Zamprônio dos Santos, Celso Moura Rebello, Cristiane do Prado

**Affiliations:** 1 São PauloSP Brasil Hospital Israelita Albert Einstein, São Paulo, SP, Brasil.

**Keywords:** Cânula, Bronquiolite, Fatores de risco, Respiração, Criança

## Abstract

**Objetivo:**

Avaliar a evolução de desfechos clínicos em crianças com bronquiolite que utilizaram cânula nasal de alto fluxo, e determinar com quanto tempo de não melhora clínica a terapia deve ser interrompida para escalonamento do tratamento para outras formas de suporte ventilatório.

**Métodos:**

Estudo observacional retrospectivo, de lactentes com bronquiolite que utilizaram cânula nasal de alto fluxo. Os pacientes foram divididos em dois grupos de estudo, de acordo com o sucesso ou não da terapêutica com cânula nasal de alto fluxo, nomeados Grupo Sucesso e Grupo Falha. Foram analisadas as principais características demográficas e variáveis clínicas, tendo sido avaliadas 30 minutos e 6 horas após o início do tratamento até a retirada da cânula nasal de alto fluxo.

**Resultados:**

Foram estudados 83 crianças; destas, 18 crianças (21,7%) falharam. Entre os pacientes que tiveram sucesso na terapia, observou-se diminuição significativa da frequência respiratória (p<0,001), e também aumento significativo da saturação de oxigênio (p<0,001) já nos primeiros 30 minutos. O Grupo Sucesso foi significativamente diferente do Grupo Falha a partir de 6 horas, tanto para frequência respiratória (p<0,01), quanto para saturação de oxigênio (p<0,01).

**Conclusão:**

Na ausência de melhora dos sinais clínicos, a falha da terapia com cânula nasal de alto fluxo já pode ser considerada a partir de 30 minutos e, no máximo, em até 6 horas após o início da terapia. Após esse período sem melhora, o escalonamento para outro tipo de suporte ventilatório deve ser avaliado.

## INTRODUÇÃO

A cânula nasal de alto fluxo (CNAF) vem sendo amplamente utilizada como suporte respiratório em pacientes com bronquiolite. O sistema conta com a entrega de altos fluxos aquecidos e umidificados, de forma não invasiva, com fração de oxigênio titulável, podendo gerar uma pressão de distensão positiva, com aumento da capacidade residual funcional e redução do trabalho respiratório.^(^^1^^,^^2^^)^

O uso dessa terapia vem sendo associado à melhora na lavagem do espaço morto nasofaríngeo do *clearance* mucociliar do pulmão, e da oferta de oxigênio mais fidedigna, em comparação com outros sistemas de entrega de oxigênio.^(^^3^^)^ A CNAF melhorou os níveis de saturação de oxigênio (SpO_2_) e está associada a uma diminuição do *end tidal* de gás carbônico (EtCO_2_) e da frequência respiratória (FR) em crianças com bronquiolite hospita­lizadas em unidades de terapia intensiva (UTI).^(^^4^^,^^5^^)^

Inicialmente, o foco dos estudos era trazer protocolos que guiassem a titulação do fluxo utilizado. Surgiram protocolos que utilizavam fluxos predeterminados por faixa etária,^(^^6^^)^ e outros que faziam a titulação do fluxo baseado no peso; atualmente, o fluxo mais utilizado varia de 1,0 a 2,0L.kg-^1^.min-^1^.^(^^7^^−^^10^^)^

Embora alguns autores tenham estudado variáveis clínicas, como frequência cardíaca (FC), FR, fração inspirada de oxigênio (FiO_2_) e SpO_2_, para determinar a falha terapêutica do CNAF, ainda não há consenso sobre os valores máximos aceitáveis.^(^^6^^−^^8^^,^^11^^,^^12^^)^ E não existem estudos sobre o tempo necessário para determinar a falha da CNAF, como já é bem estabelecido para a ventilação mecânica não invasiva (VNI), tanto na população adulta quanto na pediátrica.^(^^13^^−^^16^^)^

Durante décadas, utilizou-se a VNI para tratamento da insuficiência respiratória sem a compreensão dos limites dessa terapia, que atualmente ainda são conhecidos. Estudos relacionados à falha da VNI com necessidade de intubação orotraqueal apontaram para o fato de que pacientes que não demonstravam resultados a curto prazo (1 a 2 horas) apresentavam maior risco de falha da VNI,^(^^13^^,^^14^^)^ e o atraso na interrupção da VNI pode ter sido associado ao aumento da mortalidade.^(^^15^^,^^16^^)^ A determinação dos critérios de resultados e o prazo para interrupção da terapia da CNAF se fazem necessários para que ela possa ser usada com segurança.

## OBJETIVO

Avaliar a evolução de desfechos clínicos em crianças com bronquiolite que utilizaram cânula nasal de alto fluxo, e determinar com quanto tempo de não melhora clínica a terapia deve ser interrompida para escalonamento do tratamento para outras formas de suporte ventilatório.

## MÉTODOS

### Tipo e local do estudo

Foi realizado um estudo observacional retrospectivo, na Unidade de Terapia Intensiva Pediátrica do Hospital Israelita Albert Einstein, por meio da análise do prontuá­rio das crianças que preencheram os critérios de inclusão para o delineamento da amostra. Por se tratar de um estudo retrospectivo houve isenção do termo de consentimento livre e esclarecido (TCLE).

### Pacientes

Foram elegíveis para o estudo crianças com idade inferior a 2 anos com diagnóstico de bronquiolite, que utilizaram CNAF como primeira escolha para tratamento da insuficiência respiratória, e foram admitidas na unidade de terapia intensiva (UTI) pediátrica do Hospital Israelita Albert Einstein, na cidade de São Paulo (SP), no período de janeiro de 2016 a junho 2017.

A bronquiolite é definida como uma inflamação de pequenas vias aéreas, de etiologia viral, que acomete lactentes com idade entre zero e 2 anos. Cursa com aumento da secreção de muco e edema de via área, levando à obstrução da via aérea, com grau variável de intensidade.

As variáveis analisadas incluíam idade, sexo, peso na admissão, *Pediatric Index of Mortality* (PIM) 2, *Modified Wood’s Clinical Asthma Score* (M-WCAS), e tempo de utilização de CNAF. Para análise do desfecho clínico, os pacientes foram avaliados quanto à FR, FC, FiO_2_ e SpO_2_, nos momentos pré-instalação, 30 minutos, 6 horas e retirada da CNAF.

Para análise dos dados, os pacientes foram divididos em dois grupos: Grupo Sucesso (pacientes que melhoram com a terapia da CNAF) e o Grupo Falha (pacientes que necessitaram de outro tipo de suporte ventilatório).

### Critérios de falha da cânula nasal de alto fluxo

Os critérios para falha na terapia com CNAF ainda não estavam estabelecidos na instituição onde a pesquisa se desenvolveu. Dessa forma, a falha na terapia com CNAF se baseou na avaliação da equipe assistencial, e foi definida como necessidade de VNI ou ventilação mecânica invasiva (VMI).

### Equipamento de alto fluxo

Para realização da terapia com CNAF, foram utilizados um dos dois sistemas disponíveis na UTI pediátrica: o Optiflow™ (Fisher & Paykel Healthcare, Auckland, Nova Zelândia) ou o sistema Precision Flow^®^ – Vapotherm (New Hampshire, Estados Unidos). O sistema Optiflow™ foi utilizado associado ao *blender* Babypap^®^ 1150-S (Fanem, Guarulhos, SP, Brasil). As cânulas nasais Optiflow™ Junior 2 (Fisher & Paykel Healthcare, Auckland, Nova Zelândia) foram utilizadas em quatro tamanhos, conforme adequado para cada paciente, a saber: OPT312 Premature, OPT314 Neonatal, OPT316 Infant e OPT318 Pediatric. O sistema Precision Flow^®^ foi utilizado com as cânulas com quatro tamanhos diferentes: *neonate, infant*, *pediatric small* e *pediatric*. O protocolo de utilização foi baseado na titulação do fluxo a 2,0L.kg^−1^.min^−1^ para pacientes até 10kg; para pacientes acima de 10kg, foi utilizado fluxo de 2,0L.kg^−1^.min^−1^ até o décimo quilograma, e 0,5L.kg^−1^.min^−1^ a partir do décimo primeiro quilo, ou seja, para cada quilo acima do 10kg.

### Análise estatística

As variáveis categóricas foram descritas por frequências absolutas e percentagens. As variáveis numéricas foram verificadas quanto às suas distribuições por meio de *boxplots* e descritas por medianas e quartis, por se verificarem distribuições assimétricas e com valores discrepantes. A descrição está apresentada de forma global e também de acordo com a falha ou não na utilização da CNAF.

Para comparar o perfil dos pacientes com ou sem falha na utilização da CNAF, foram utilizados testes de hipóteses: teste do χ^2^, teste exato de Fisher e teste de Mann-Whitney, conforme adequado.

Para investigar variações nas medidas obtidas no decorrer do tempo, foram ajustados modelos mistos, que consideraram a dependência entre medidas obtidas em diferentes momentos em um mesmo indivíduo. Foram consideradas distribuições gama, por serem mais apropriadas a dados assimétricos contínuos. Os resultados foram expressos por valores médios estimados, intervalos de confiança de 95% (IC95%) e valores p.

Nos casos de comparações múltiplas, os valores de p foram corrigidos pelo método de Bonferroni sequencial.

As análises foram realizadas com o programa (SPSS), considerando nível de significância 5%.

O trabalho foi submetido ao Comitê de Ética em Pesquisa institucional e está registrado sob o número 2.316.087, CAAE: 77279317.4.0000.0071.

## RESULTADOS

Das 1.749 crianças admitidas no período do estudo, 363 (20,8%) tinham diagnóstico principal de bronquiolite. Destas, 83 crianças utilizaram CNAF. A falha do CNAF ocorreu em 18 crianças (21,7%), sendo que cinco pacientes (6,0%) precisaram de ventilação mecânica (VM) e 17 pacientes (20,5%) precisaram de VNI; quatro deles precisaram tanto de VM quanto de VNI.

A tabela 1 apresenta as principais características demográficas, intervenções clínicas e desfechos tardios dos pacientes de forma global e de acordo com sucesso ou falha no uso de CNAF. Não houve diferença entre os grupos em relação às características demográficas. Na análise do tempo total de utilização da CNAF, pacientes no Grupo Falha utilizaram a terapia por menos tempo quando comparado ao Grupo Sucesso: 12,8 horas *versus* 56,8 horas (p<0,001).

**Tabela 1:** Características demográficas dos pacientes em geral e de acordo com sucesso ou falha da terapia com cânula nasal de alto fluxo

Variáveis		Total (n=83)	Grupo		Valor de p
			Sucesso (n=65)	Falha (n=18)	
Idade, meses		2,00 (1,00-6,00)	3,00 (1,00-6,00)	2,00 (1,25-3,00)	0,55*
Sexo					
	Masculino	46 (55,4)	39 (60,0)	7 (38,9)	0,18†
	Feminino	37 (44,6)	26 (40,0)	11 (61,1)	
Peso na admissão, kg		5,70	5,80	4,95	0,11*
Escore PIM 2, gravidade (0-100%)		0,16	0,16	0,16	0,73*
Escore M-WCAS		4,00	4,00	4,00	0,50*
Tempo uso CNAF, horas		4,00 (25,87-70,50)	56,8 (40,00-74,67)	12,8 (8,87-23,69)	<0,001*

Valores expressos por mediana (intervalo interquartil) ou n (%).

* teste Mann-Whitney;

† valores de p para teste do χ^2^.

PIM: *Pediatric Index of Mortality*; M-WCAS: *Modified Wood’s Clinical Asthma Score*; CNAF: cânula nasal de alto fluxo.

Na tabela 2, foram apresentadas as médias estimadas pelos modelos ajustados para as medidas de FR, FiO_2_ e SpO_2_ comparando os momentos pré-instalação, 30 minutos pós-instalação, 6 horas pós-instalação e retirada da CNAF, também comparando os Grupos Sucesso e Falha.

**Tabela 2:** Valores médios ajustados e intervalos de confiança de 95% para as medidas obtidas nos momentos: pré, 30 minutos, 6 horas e retirada da cânula nasal de alto fluxo

	Grupo Sucesso (n=65)				Grupo Falha (n=18)			
	Pré-CNAF	30 minutos	6 horas	Retirada	Pré-CNAF	30 minutos	6 horas	Retirada
FR (irpm)	54,6 (52,2-57,1)	48,4 (46,4-50,4)*	45,6 (43,7-47,5)*	39,7 (37,8-41,6)*	59,5 (54,6-64,5)	52,5 (48,5-56,5)	51,9 (47,9-55,9)	59,7 (54,9-64,4)
FiO_2_ (%)	37,1 (33,6-40,6)	31,0 (28,6-33,5)†	28,8 (27,0-30,7)*	24,2 (22,7-25,6)*	34,9 (28,7-41,0)	29,8 (25,3-34,3)	30,9 (27,1-34,6)	33,8 (30,6-37,0)
SpO_2_ (%)	94,1 (92,8-95,4)	97,0 (96,5-97,5)*	97,2 (96,7-97,8)*	97,2 (96,3-98,1)*	94,9 (92,4-97,4)	97,7 (96,7-98,7)	95,6 (94,4-96,7)	92,8 (91,1-94,5)

* p<0,001 em relação ao valor pré do Grupo Sucesso;

† p<0,01 em relação ao valor pré do Grupo Sucesso.

CNAF: cânula nasal de alto fluxo; FR: frequência respiratória; irpm: incursões respiratórias por minuto; FiO_2_: fração inspirada de oxigênio; SpO_2_: saturação periférica de oxigênio.

Em geral, observou-se que, para os pacientes que tiveram sucesso na terapia com CNAF, houve queda significativa nas medidas de FR e FiO_2_ já nos primeiros 30 minutos (p<0,001), bem como aumento significativo da SpO_2_ também nos primeiros 30 minutos de uso da cânula (p<0,001). Pacientes com sucesso na CNAF foram significativamente diferentes do Grupo Falha a partir de 6 horas, tanto para FR (p<0,01) quanto para SpO_2_ (p<0,01).

## DISCUSSÃO

A literatura mostra grande avanço no entendimento sobre a utilização da CNAF na população pediátrica, especificamente em lactentes com bronquiolite, porém, o critério de falha com necessidade de escalonamento da terapêutica ainda não é claro.^(^^11^^−^^12^^,^^17^^−^^21^^)^

Até o momento, poucos estudos observaram variáveis clínicas para determinação da falha da terapia com CNAF.^(^^17^^−^^21^^)^ Este foi um dos estudos pioneiros a estabelecer um prazo para interrupção da terapia quando observada a não melhora do paciente, além de determinar parâmetros clínicos. A determinação desses parâmetros e de seu comportamento entre o início da terapia e sua interrupção traz segurança na utilização da CNAF, uma vez que determina precocemente a não resposta ao tratamento, evitando deterioração do sistema respiratório.

Neste estudo, os pacientes que falharam na terapêutica com o CNAF não apresentaram melhora clínica significativa com redução da FR e da FiO_2_, e aumento da SpO_2_ a partir de 30 minutos após o início da terapia, o que foi observado nos indivíduos com sucesso na terapêutica. Demonstrou-se, também, que foi possível encontrar diferença significativa na FR e na SpO_2_ entre os pacientes que tiveram sucesso e os que falharam a partir de 6 horas.

Mayfield et al., corroboram os resultados deste estudo, pois também mostraram melhora na FR entre os pacientes que responderam à terapia com CNAF após 1 hora de tratamento. No entanto, o número de pacientes incluídos nesse estudo foi muito menor que na presente pesquisa: apenas oito pacientes no grupo de falha.^(^^9^^)^

Outros estudos também evidenciaram a não melhora da FR e da oxigenação entre pacientes que falharam à terapia, porém sem discutir o tempo para interrupção.^(^^17^^−^^19^^)^

Embora o comportamento de variáveis como FC, FR e FiO_2_ após a adaptação da CNAF esteja relacionado ao sucesso ou à falha da terapia, não há consenso quanto ao tempo necessário para reavaliar o paciente, determinar que não houve melhora e interromper a terapia com CNAF. Estudos mostram tempo bastante variável para interrupção e escalonamento para VNI ou VMI, que podem ser de 12,8 horas, como no presente estudo, 5,5 horas,^(^^2^^0)^ 7 horas^(^^18^^)^ e 14 horas.^(^^20^^)^ Isso traz algumas considerações em relação à vigilância desses pacientes em utilização de CNAF e mostra que a percepção de não melhora e o escalonamento para outro tipo de terapia podem ser mais precoces.

Os achados deste estudo mostram que os pacientes que tiveram sucesso com a CNAF responderam rápido (30 minutos) e que, com 6 horas de utilização, já há diferença significante na FR e na SpO_2_ entre os grupos (p<0,01). Os resultados do presente estudo sugerem que pacientes que não responderam à terapia com 6 horas de utilização, permanecendo com manutenção da taquipneia e necessidade de FiO_2_ em geral maior do que 30% para alcance de SpO_2_ >95%, devem ser escalonados para outras formas de terapia. Pesquisas apontam^(^^17^^,^^18^^,^^20^^)^ a interrupção da terapia com tempos superiores há 6 horas, e, na prática clínica, alguns pacientes permanecem até 24 horas para decisão da interrupção pelo time assistencial. Os achados deste estudo, nesse sentido, contribuem para o escalonamento precoce da terapia e a segurança do paciente.

Nesta pesquisa, foi observada falha da terapia com CNAF em 21,7% dos casos, sendo que cinco deles necessitaram de intubação orotraqueal. A literatura mostra grande variabilidade na percentagem de falha da terapia de alto fluxo em pacientes com bronquiolite (de 0% a 50%).^(^^17^^,^^21^^−^^23^^)^ Milési et al., ao compararem a CNAF com a pressão positiva contínua das vias aéreas (CPAP) nasal em pacientes com bronquiolite, observaram 50% de falha no grupo com CNAF.^(^^21^^)^ O perfil de pacientes daquele estudo em relação ao M-WCAS era bastante semelhante ao dos pacientes deste trabalho. Apesar disso, aqui a taxa de falha foi inferior à encontrada por Milési et al., 21,7% *versus* 50%.^(^^21^^)^ Esse fato pode ser explicado pela diferença da taxa de fluxo inicial entre o estudo de Milési et al., e presente, que é de 1,0L.kg^−1^.min^−1^ *versus* 2,0L.kg^−1^.min^−1^.^(^^21^^)^

Franklin et al., em estudo comparando CNAF com cânula nasal de baixo fluxo em pacientes com bronquiolite, observaram 10% de falha da CNAF, enquanto 61% dos pacientes que falharam na terapia de baixo fluxo foram resgatados com sucesso após iniciar CNAF.^(^^23^^)^ A alta variabilidade na percentagem de falha da CNAF é apresentada pela literatura^(^^17^^,^^21^^−^^23^^)^ por diversos fatores. O perfil dos pacientes incluídos é um desses fatores, já que quadros leves com escore de desconforto menor têm maior chance de responder à terapia, enquanto quadros graves com escore de desconforto maior têm maior chance de falha. O local de estudo é outro fator, quando se compara UTI a enfermarias. Além disso, é possível que a falta de padronização do fluxo-alvo e a utilização de métodos subjetivos de avaliação para determinar a falha da terapia dificultem a comparação da percentagem de falha nos diversos estudos.

Este estudo tem as limitações implícitas de um trabalho retrospectivo, apesar de não haver viés na forma de aplicação da CNAF, pois o protocolo de utilização da CNAF já estava bem definido na época do levantamento dos casos. Outra limitação é o pequeno número de pacientes que falharam com a CNAF durante o período. Embora 83 crianças tenham sido incluídas, apenas 18 crianças falharam com a CNAF, o que limitou o poder das análises. Até o momento, estudos avaliando fatores de risco para falha na terapia com CNAF mostraram perfil semelhante ao encontrado nesta pesquisa, com 14 crianças no grupo com falha no estudo de Betters et al.,^(^^20^^)^ e oito no de Mayfield et al.^(^^9^^)^

## CONCLUSÃO

Na ausência de melhora dos sinais clínicos, como redução da frequência respiratória e da fração inspirada de oxigênio, e do aumento da saturação de oxigênio, a falha da cânula nasal de alto fluxo já pode ser considerada a partir de 30 minutos e, no máximo, até 6 horas após o início da terapia. Após esse período sem melhora, deve ser avaliado o escalonamento para outro tipo de suporte ventilatório.
